# Exome sequencing helped the fine diagnosis of two siblings afflicted with atypical Timothy syndrome (TS2)

**DOI:** 10.1186/1471-2350-15-48

**Published:** 2014-04-29

**Authors:** Sebastian Fröhler, Moritz Kieslich, Claudia Langnick, Mirjam Feldkamp, Bernd Opgen-Rhein, Felix Berger, Joachim C Will, Wei Chen

**Affiliations:** 1 Berlin Institute for Medical Systems Biology, Max-Delbrück-Center for Molecular Medicine, Robert-Rössle-Str. 10, Berlin 13125, Germany; 2 Division of Pediatric Cardiology, Charité University Hospital, Augustenburger Platz 1, Berlin 13353, Germany

**Keywords:** Timothy syndrome, Exome sequencing, Mosaic mutation, LQTS

## Abstract

**Background:**

Long-QT syndrome (LQTS) causes a prolongation of the QT-interval in the ECG leading to life threatening tachyarrhythmia and ventricular fibrillation. One atypical form of LQTS, Timothy syndrome (TS), is associated with syndactyly, immune deficiency, cognitive and neurological abnormalities as well as distinct cranio-facial abnormalities.

**Case presentation:**

On a family with both children diagnosed with clinical LQTS, we performed whole exome sequencing to comprehensively screen for causative mutations after a targeted candidate gene panel screen for Long-QT syndrome target genes failed to identify any underlying genetic defect. Using exome sequencing, we identified in both affected children, a p.402G > S mutation in exon 8 of the CACNA1C gene, a voltage-dependent Ca^2+^ channel. The mutation was inherited from their father, a mosaic mutation carrier. Based on this molecular finding and further more careful clinical examination, we refined the diagnosis to be Timothy syndrome (TS2) and thereby were able to present new therapeutic approaches.

**Conclusions:**

Our study highlights the difficulties in accurate diagnosis of patients with rare diseases, especially those with atypical clinical manifestation. Such challenge could be addressed with the help of comprehensive and unbiased mutation screening, such as exome sequencing.

## Background

Long-QT syndrome [1,2] (LQTS) is a cardiac chanellopathy characterized by QT prolongation and T-wave abnormalities in the ECG causing syncope, life threatening arrhythmia and sudden cardiac death due to *torsade de pointes* (TdP) and ventricular fibrillation.

On a molecular level, most LQTS patients have mutations in a set of ten genes (KCNQ1, KCNH2, SCN5A, KCNE1, KCNE2, CAV3, SCN4B, AKAP9, SNTA1 and KCNJ5) with ~97% of all mutations within this gene panel falling into the first three genes [3]. The remaining seven genes represent some rare cases of LQTS. However, in about 25% of all studied families showing LQTS symptoms, no mutations within the set of 10 candidate genes can be found [3], indicating either a limitation in the testing methods employed on the identified candidate genes or the presence of additional genes related to LQTS.

A distinct form of LQTS is the syndactyly related TS (also called LQT8) a rare autosome dominant disorder. The first clinical cases were described as the ‘heart-hand syndrome’ in the 1990’s [4-6] with a prolonged QT-interval and syndactyly being present in 100% of described cases. Additional cardinal features of TS include: bradycardia, AV-Block, congenital heart disease, immune deficiency, hypoglycemia, neurological and cognitive abnormalities such as intellectual disability, autism and seizures as well as distinctive cranio-facial abnormalities. Also, an association of TS with hypertrophic cardiomyopathy has been described [7].

In 2004, a missense mutation (p.406G > R) located in exon 8A of the gene *CACNA1C*, encoding the transmembrane segment S6 of domain I of CaV1.2 (Voltage-dependent L-type calcium channel subunit alpha-1C), has been identified to cause TS [8]. The mutation leads to almost complete loss of voltage dependent channel-inactivation, thereby resulting in intracellular calcium overload and subsequently delayed repolarization of the respective cell. The vast majority of described cases result from de-novo missense mutations. Nevertheless, in one family with two affected children, mosaicism for the p.406G > R mutation was discovered in the mother [8]. In 2005, Splawski et al. described two unrelated individuals with severe prolongation of the QTc-interval without syndactyly, in which two de novo mutations (p.406G > R and p.402G > S) in Exon 8 of *CACNA1C* have been identified to cause a related but distinct phenotype, classified as Timothy syndrome 2 (TS2) [9]. In contrast to the splice variant containing Exon 8A, Exon 8 is widely expressed in heart and brain, consistent with the phenotype of the described patients. Whereas patient one, described by Splawski, presented with severe central nervous system (CNS) and cardiac affection, patient two presented with severe QTc prolongation but milder CNS affection which was attributed to the somatic mosaicism for the mutation.

In this report, we describe a family with two affected children initially diagnosed to have LQTS. After a candidate gene panel test for LQTS failed to identify any causative genetic defect, we referred to whole-exome sequencing and identified a causative p.402G > S (exon 8) mutation in the *CACNA1C* gene in both affected children. The mutation was inherited from the father, who is mosaic for the specific mutation. As a result, we refined the diagnosis to be Timothy syndrome type 2 (TS2) although both patients do not manifest the full clinical spectrum of classical TS2. On one hand, our study highlights the difficulties in accurate diagnosis of patients with rare diseases, especially those with atypical clinical manifestation. On the other hand, we demonstrated such challenge could be addressed with the help of comprehensive and unbiased mutation screening, such as exome sequencing.

## Case presentation

### Patients

**Figure 1:**
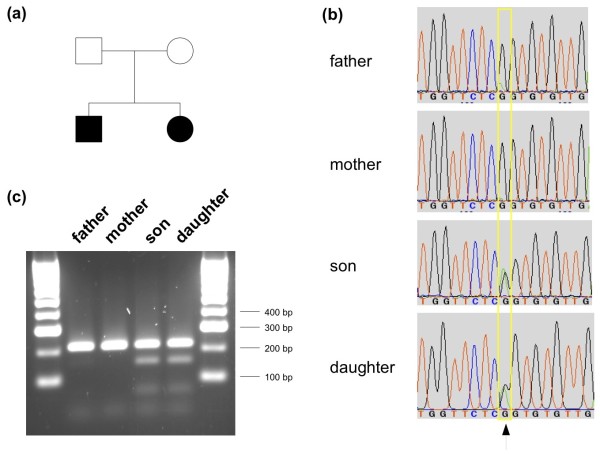
**Pedigree and validation results for the causative CACNA1C G402S mutation. (a)** Pedigree of the Lebanese family. Blackened symbols indicate the affected individuals. **(b)** Sanger sequencing traces validating the *CACNA1C* c.1204G > A mutation in both affected children as well as the mosaic mutation in the father. **(c)** Restriction assay using enzyme DdeI, specifically cleaving PCR amplicons carrying the c.1204G > A mutation. A clear restriction pattern was found for both affected children, no restriction pattern was found for the apparently healthy mother and a mosaic restriction pattern was found for the father.

The family is originated from Lebanon with two children of non-consanguineous healthy parents (Figure 1a). Family history is negative for cases of sudden cardiac death (SCD), arrhythmia, LQTS or gross neurological abnormalities. Electrocardiograms of both the father (who was found to be a p.G402S mosaic variant carrier) and the mother are unremarkable and show a normal QTc-interval. The father’s 6 siblings who live in Lebanon were not investigated (no ECGs). Both children present with significantly prolonged QTc-intervals (Patient 1 468–547 ms, Patient 2 476–650 ms), T-wave alternans and recurring life threatening arrhythmia since early childhood. After receiving the molecular diagnosis, mild partial syndactyly of the second/third toe, which usually is regarded as a normal variant, was found in both patients as well as in their father. It is speculative whether this finding could be related to the p.402G > S mutation, since the few reported patients carrying this mutation do not show syndactyly [9]. Both children do not meet the full clinical criteria for classical TS apart from the prolonged QTc interval: Their hearts are structurally and functionally normal, they do not show any cranio-facial dysmorphies, they do not suffer from recurrent infections and no other major anomalies as manifested in TS (type 1) can be detected (see Additional file 1).

The index patient is the 8 year old daughter (Patient 1) who presented VF/ TdP leading to cardiac arrest and was successful resuscitated at the age of 2 months. Antiarrhythmic therapy was started with beta blockers, nicorandil and mexiletine, controlling TdP best with high doses of propranolol and mexiletine. At the age of 4, her parents agreed to have an internal cardiac defibrillator (ICD) implanted, who delivered 2 adequate shock since then. The patient developed generalized epilepsy which is currently well controlled with levetiracetam. Today, at eight years of age, the patient is unable to speak and learned walking only at the age of seven. She additionally shows evidence of a vast precocious puberty which is not known to be associated with TS. The neurological findings (epilepsy, developmental delay) are rather attributed to hypoxic brain damage caused by cardiac arrest and resuscitation than being part of TS. Especially since the early neonatal development of the patient has been entirely unremarkable. Moreover, patient 2 unlike his sister reached the milestones of psychomotor development at the expected times and clinical workup failed to show any neurological abnormalities. He is the second child of the family and is now six years old. He developed VF/ TdP at the age of 3 years and was successfully resuscitated using his sister’s automatic external defibrillator and received an ICD two months later. As his sister, he was put on propranolol and mexiletine. Since then, the patient has had frequent episodes of VF and non-sustained ventricular tachycardia and has received overall more than 40 adequate and appropriate shocks. His antiarrhythmic therapy has been modified several time, he was administered several antiarrhythmic drugs (AAD): high-dose betablockers, potassium-, magnesium- and calcium-antagonists as well as Vaughn-Williams class Ia, Ib and Ic agents. None of these AAD combinations shortened QTc-intervals or reduced VF episodes. Left- and right-cardiac sympathetic denervation could also not reduce the arrhythmic episodes.

Today, quality of life of both patients is still significantly affected by the continuing relapses of VF, especially in the boy, who seems to be especially insusceptible to standard therapeutic approaches.

Written informed consent was obtained from each family member for the subsequent studies according to the German Genetic Diagnostic Law (GenDG).

### Candidate gene panel screening for LQTS

Given the diagnosis of LQTS for both children, LQTS candidate gene panels I, II and III, including genes: *KCNQ1*, *KCNH2*, *SCN5A*, *KCNE1* and *KCNE2* were PCR amplified and Sanger-sequenced to identify mutations. Neither this screen, nor a multiplex ligation probe amplification (MLPA) of 40 regions of the target genes identified any mutations or insertions/ deletions in the regions screened (both analysis were performed at the Center for Human Genetics and Laboratory Medicine, Martinsried, Germany).

### Exome sequencing and bioinformatics

All four family members were subjected to exome sequencing. Genomic DNA was isolated from blood samples using standard methods. Five micrograms of genomic DNA were enriched using the Agilent Human All Exon V4 kit (Agilent Technologies, Santa Clara, CA, USA) following the manufacturer's protocol. A list of screened genes and a list of assayed regions for these probes is available on the Agilent website (https://earray.chem.agilent.com/suredesign/).

Whole-exome libraries were sequenced on an Illumina HiSeq 2000 system for 1x101 cycles following the manufacturer's instructions (Illumina, San Diego, CA, USA). All raw sequencing reads were mapped onto UCSC hg19 [10] using BWA 0.5.9-r16 [11], mappings were converted into BAM file format using samtools 0.1.18 [12]. Initial mappings were post-processed using GATK 1.3-21 [13] following their ‘best practices V3’. In brief, reads were realigned around sites of known insertions and deletions (INDELs). Then, likely PCR duplicates were detected using Picard 1.48 [12]. Finally, raw base quality scores were empirically recalibrated. Single Nucleotide Polymorphisms (SNPs) and short INDELs were identified using the UnifiedGenotyper from GATK and in parallel using the variant caller included in samtools. Variants were classified as novel or known variants deposited in dbSNP 135 [14]. Functional consequences of each variant were annotated using snpEff 2.0.5d [15] for UCSC hg19 RefSeq genes and ENSEMBL 65 human gene models [10,16]. The potential deleterious effect was evaluated using PolyPhen 2 [17], SIFT [18], PhyloP [19], MutationTaster [20], GERP++ [21], LRT [22] and OMIM [23] if available. Candidate variants from GATK and from samtools were compared to increase both sensitivity and specificity.

### Mutation validation

To confirm the presence and identity of the p.402G > S variant in *CACNA1C* exon 8, Sanger sequencing was performed on PCR amplicons from genomic DNA covering that variant position. A restriction assay on the same amplicons using the enzyme DdeI that specifically cleaves amplicons carrying the mutation was also performed to validate the mosaicism in the father.

### Exome sequencing

All four family members were subjected to whole exome sequencing. In total, we obtained 215 – 218 million single-end 101 bp reads per sample, of which 98.5 – 98.7% could be mapped onto the human genome. After removing duplicated reads, which possibly derived from PCR artifacts, 48 – 50 million unique reads were mapped to the targeted protein coding regions, resulting in an average of 96.9-101.2x coverage within the targeted coding region (Table 1).

**Table 1:** Summary statistics for exome sequencing

	**All reads**	**# mapped reads**	**# uniquely mapped reads**	**# non-redundant uniquely mapped reads**	**# non-redundant reads uniquely mapped in targeted regions**	**Average sequencing depth in target regions**
**Father**	215,270,552	212,495,384	207,507,067	88,105,506	49,357,904	100.29
**Mother**	218,495,007	215,280,440	210,261,330	84,480,091	47,828,952	97.15
**Son**	217,253,295	214,298,717	209,188,980	85,277,852	47,758,981	96.87
**Daughter**	218,048,872	214,904,784	209,988,667	89,229,385	49,872,405	101.19
**Mean**	217,266,932	214,244,831	209,236,511	86,773,209	48,704,561	98.88
**sd**	1,426,571	1,234,435	1,239,520	2,258,488	1,072,618	2.19

Using GATK and samtools, we detected 26,501 – 30,024 SNPs and INDELs in the exome of each family member, of which 94.3 – 97.1% are known variants deposited in dbSNP 135 (see Additional file 2). Given the pedigree, we first searched for non-silent variants present in both affected children but absent in both apparently healthy parents. Whereas no such variants were found by GATK, samtools outputs one such variant in exon 8 of the gene *CACNA1C* (c.1204G > A, p.402G > S).

We then checked for the reason leading to the inconsistent results from samtools and GATK. While both tools called the same genotypes in affected siblings as well as their mother, the father was identified to carry heterozygous or homozygous reference allele by GATK (PHRED-scaled genotype quality 99) and samtools (PHRED-scaled genotype quality 50), respectively. After retrieving the original mapping data from the father, we found 15 high-quality reads (9.9% of all 151 reads aligned at this position) carrying the variant allele. This relatively low non-reference allele frequency, indicating a mosaic carrier, allowed GATK to call a heterozygous genotype, whereas it does not pass the default samtools’ variant calling threshold.

### Mutation validation

Sanger sequencing on the blood samples of all family members not only confirmed the heterozygous p.402G > S variant in both affected children but also the mosaic genotype for this variant in the father (Figure 1b). The father's mosaic genotype was also observed in his oral mucosa swap sample at a similar allele frequency (see Additional file 3). Both heterozygous and mosaic genotypes could also be validated by a restriction assay using DdeI, specifically cleaving amplicons containing the variant (Figure 1c).

### Refined diagnosis and treatment

The p.G402S variant in exon 8 of the gene *CACNA1C* has been previously associated with Timothy Syndrome TS2 (MIM: 601005). In literature, treatment with multipotent ion-channel-blocker ranozaline in combination with verapamil has been reported to be beneficial in a patient with this distinct mutation [24]. This therapeutical approach is currently applied in our patients with short follow up time.

Cell-culture-based experiments with Cav1.2. and Timothy channels show that calcineurin inhibitors (e.g. FK 506, cyclosporine A) stabilize the action potential of the respective cells by increasing voltage dependent channel-inactivation [25]. Theoretically this could be another therapeutical approach in case of TS refractory to conventional medical therapy.

Using whole-exome sequencing we identified a p.402G > S mutation in exon 8 of the gene *CACNA1C* in a family with two affected children, both presenting atypical form of Timothy syndrome (TS2). This variant has been previously described to alter the transmembrane segment S6 of domain I in the *CACNA1C* gene, thereby almost completely disrupting voltage-dependence of this Ca^2+^-channel, leading to a massively increased QT-interval in an electrocardiogram [9]. The *CACNA1C* isoform containing exon 8 is reported to be the predominant isoform expressed in heart and brain, consistent with the phenotype of both patients.

A previous LQTS candidate gene panel screen returned no diagnostic result since, at the time of evaluation, both children only unequivocally manifest the LQTS part of the Timothy Syndrome phenotype, together with an only partial syndactyly that they even share with their father - a feature quite easy to oversee by a cardiologist focusing on their severe heart arrhythmia. Exome sequencing, as an unbiased diagnostic aid however, clearly identified the causative mutation after a careful analysis.

The p.402G > S mutation was inherited from the mosaic father. However, a mosaic genotype, manifested with a relatively low non-reference allele frequency in the exome sequencing results is not susceptible to most variant calling algorithms. Interestingly, although samtools lead to the discovery of this mutation, a (somehow) wrong genotype call was made by samtools, whereas the correct genotype call of GATK resulted in filtering out this variant from the list of autosome-dominant events, given the father is not afflicted with Timothy syndrome. Luckily, mosaicism is a rare event and most studies will identify the same causative mutation using different variant callers. However, care should be taken in case of pedigrees with an atypical inheritance pattern where a combination of variant callers with different genotyping models/thresholds can provide a valuable list of discordant genotype calls, thereby enriching for candidate mosaicisms. Those variants might just be filtered out when relying on a single variant caller.

Diagnosing patients with rare diseases is a complex mission that can easily take several years of clinical examinations. Many different gene defects can give rise to clinically indistinguishable phenotypes. Recent data show that up to 40% of all initial diagnosis are wrong [26,27]. Current whole-exome/whole-genome sequencing technology, applied in clinical settings, can aid in correctly diagnosing those patients, as demonstrated in this study. With the ever decreasing sequencing costs, a global, truly unbiased genomic screen, at a cost even below that of targeted gene panel screens today, will be available in the clinic in only a few years. Combined with a genotype-phenotype-treatment resource, constantly expanding during such kind of studies in the clinic, new therapeutic approaches could be offered to the patient as an immediate benefit. However, in such clinical context, controlled vocabularies for phenotyping patients together with rigid data protection strategies must be warranted at all times. Additional legislative effort might be required for recording, storing and accessing this kind of data. Furthermore, a clear consent must be obtained regarding how to proceed with incidental findings. Whether those should be conveyed to the patient if requested, whether to only convey results on ‘treatable diseases’ or even only to convey findings immediately relevant for the disease studied must be agreed upon before initiating such kind of studies. Finally, diagnosing a ‘syndrome’ might stigmatize the patient, so proper genetic counseling by specialized physicians will be required for all such molecular genetic diagnostics.

## Conclusions

We have described a family with two affected children initially diagnosed to have LQTS. After a candidate gene panel screen for LQTS failed, we referred to whole-exome sequencing of the whole family as a global, unbiased method. As a result, we have identified a causative p.402G > S mutation in the *CACNA1C* gene in both affected children and showed that this mutation was inherited from their mosaic father. Thereby, we could refine the diagnosis for both children to be Timothy Syndrome (TS2), a very rare atypical form of LQTS. This refined diagnosis immediately opened new therapeutic options for the patient. Our study strongly supports the benefit of exome sequencing in clinical diagnosis of complex cases and soon might become a widely used tool in clinical settings.

## Competing interests

The authors declare no conflict of interest.

## Authors’ contributions

JCW diagnosed LQTS in the second child (child one was diagnosed before in Cologne, Germany) and is in charge of both patient's treatment for several years. MK initiated genetic workup using Next-Generation Sequencing by obtaining blood DNA samples from the family for exome sequencing, for which CL and MF performed all wet-lab experiments and SF performed all data analysis. SF and WC interpreted the results, identified the candidate mutation and drafted the manuscript. All authors read and approved the final manuscript.

## Pre-publication history

The pre-publication history for this paper can be accessed here:

http://www.biomedcentral.com/1471-2350/15/48/prepub

## Supplementary Material

Additional file 1 **Table S1.** Clinical features of Timothy Syndrome in the patients. Phenotypic features of Timothy Syndrome (TS1, TS2), as well as Patient 1 and 2 listed in the table; *:CNS features in patient 1 can be in interpreted as neurological sequelae after resuscitation (details see text). Clinical features present in Timothy syndrome type 1 patients (%) and those that have been reported in Timothy syndrome type 2 were extracted from: [8,9]. Click here for file

Additional file 2 **Table S2.** Variant calling statistics for GATK and samtools. Click here for file

Additional file 3 **Figure S1.** Validation of the CACNA1C SNV and the mosaic genotype in the father from an oral mucosa swap sample. Click here for file
