# Supplementary material for: Exome sequencing helped the fine diagnosis of two siblings afflicted with atypical Timothy syndrome (TS2)
Source: BMC Med Genet. 2014 Apr 29;15:48. doi: 10.1186/1471-2350-15-48 (PMC4038115; doi:10.1186/1471-2350-15-48)
Supplement: Additional file 1 — Table S1. Clinical features of Timothy Syndrome in the patients. Phenotypic features of Timothy Syndrome (TS1, TS2), as well as Patient 1 and 2 listed in the table; *:CNS features in patient 1 can be in interpreted as neurological sequelae after resuscitation (details see text). Clinical features present in Timothy syndrome type 1 patients (%) and those that have been reported in Timothy syndrome type 2 were extracted from: [8,9]. [file 1471-2350-15-48-S1.doc]

| **Phenotype** | | **Patient 1**  **(daughter)** | **Patient 2**  **(son)** | **TS type 1**  **%** | **Reported in TS type 2** |
| --- | --- | --- | --- | --- | --- |
| **QTc prolongation** | | yes | yes | 100 % | yes |
| **Syndactyly** | | partial | partial | 100 % | no |
| **CNS features** |  | | | | |
| *Mental retardation* | | yes* | no | 25 % | yes |
| *Seizures* | | yes* | no | 21 % | yes |
| *Developmental delay of language* | | yes* | no | 62 % | yes |
| *Developmental delay of gross motor* | | yes* | no | 57 % | yes |
| *Developmental delay of fine motor* | | yes* | no | 38 % | yes |
| **Cranio-facial features** |  | | | | |
| *Flat nasal bridge* | | no | no | 83 % | yes |
| *Large cranium* | | no | no | 14 % | yes |
| **Recurrent infections** | | no | no | 75 % | yes |
