# Supplementary figures and images for: Exome sequencing helped the fine diagnosis of two siblings afflicted with atypical Timothy syndrome (TS2)
Source: BMC Med Genet. 2014 Apr 29;15:48. doi: 10.1186/1471-2350-15-48 (PMC4038115; doi:10.1186/1471-2350-15-48)

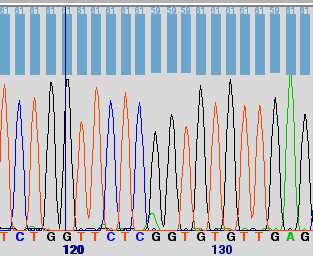


Father (forward)


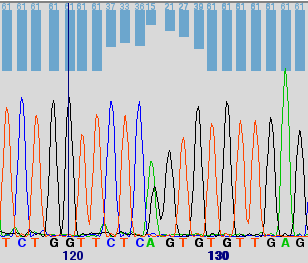


Son (forward)


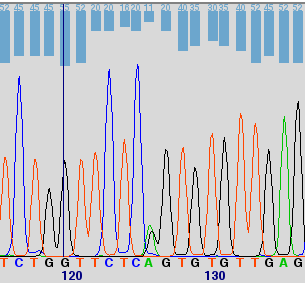


Daughter (forward)

Supplement: Additional file 3 — Figure S1. Validation of the CACNA1C SNV and the mosaic genotype in the father from an oral mucosa swap sample. [file 1471-2350-15-48-S3.doc]
